# Behavioral Structure, Activity Rhythm and Relationships to the Conservation and Welfare of Tibetan Fox in Tibet Plateau of China

**DOI:** 10.3390/vetsci13090968

**Published:** 2026-09-16

**Authors:** Xiping Wang, Xiaoping Lu, Jiayi Wu, Jinjin Wang, Zhaxi Sangzhu, Xiuxiang Meng

**Affiliations:** 1 School of Ecology and Environment, Renmin University of China, Beijing 100872, China; 2 School of Environment and Resource, Xichang University, Xichang 615013, China; 3 School of Ecology and Environment, Tibet University, Lhasa 850000, China

**Keywords:** Sanjiangyuan National Park, Tibetan fox, activity rhythm, human disturbance, infrared camera monitoring

## Abstract

As a keystone species in alpine ecosystems, the Tibetan fox (*Vulpes ferrilata*) plays an important role in maintaining ecological processes on the Qinghai–Tibet Plateau. Understanding its behavioral structure, activity rhythm, and responses to road-related human disturbance is essential for effective conservation and management. This study was conducted in the Tangbei Area of Sanjiangyuan National Park from May to December 2024 using infrared camera trapping. In total, 1166 effective camera-trap days yielded 748 independent photographs, 340 video clips, and 217 valid behavioral records. An ethogram was constructed, and activity patterns and responses to human disturbance were analyzed. The results showed that: (1) Tibetan fox behavior was dominated by locomotion (55.18%), followed by vigilance (23.20%) and scent marking (17.68%). (2) The species exhibited pronounced nocturnality, with an overall daily activity rate of 76.20%. Activity peaked at night (22:00–04:00), reaching 92.63%, while the lowest activity level occurred around midday (12:00), with an activity rate of 44.63%. (3) Roads and human activities significantly altered the behavioral structure of Tibetan foxes. The study indicates that Tibetan foxes adapt to human disturbance through temporal allocation and spatial avoidance, which may affect its social structure and increase energy expenditure. In future conservation practices, focus should be placed on the management of human activities in the region, with enhanced efforts in the construction of ecological corridors, restoration of habitat connectivity, and control of traffic intensity. This study provides scientific evidence to support wildlife conservation and human activity management in alpine protected areas of the Qinghai–Tibet Plateau. The quantified behavioral baseline and disturbance response patterns also offer reference indicators for conservation veterinary medicine assessment and wild canid welfare monitoring, which can be used to evaluate the health status of Tibetan fox populations under anthropogenic pressure.

## Simple Summary

The Tibetan fox is an endemic and keystone species of the Qinghai–Tibet Plateau, yet its behavior and responses to human disturbance remain poorly understood. Using infrared camera trapping in the Tangbei Area of Sanjiangyuan National Park from May to December 2024, we quantified the behavioral structure, activity rhythm, and behavioral responses to road traffic of wild Tibetan foxes. We found that the behavior of Tibetan foxes was dominated by locomotion, followed by vigilance and scent marking, and that the species was strongly nocturnal, with activity peaking at night (22:00–04:00) and reaching its lowest level around midday. Road traffic and human activities significantly altered their behavioral structure: in areas with high traffic disturbance, Tibetan foxes showed more locomotion and less scent marking, indicating that they adapt to human disturbance through temporal allocation and spatial avoidance. Such behavioral changes may increase energy expenditure and disrupt social communication, with implications for population health and animal welfare. Our findings provide scientific evidence for traffic management, the construction of ecological corridors, and the use of behavioral indicators in conservation veterinary medicine and wildlife welfare assessment.

## 1. Introduction

Animal behavior refers to the sequential combination of actions and postures exhibited by animals to meet survival and adaptation needs in specific environments [1]. The classification and definition of behaviors and behavioral categories in animal behavior research are inherently subjective [2]. Through systematic coding and classification frameworks, the construction of an animal ethogram can provide quantitative observation indicators for animal behavior identification and research, enhancing the repeatability and scientific reliability of behavioral observations [3,4].

As a fundamental ecological attribute of species, animal activity rhythm reflects the adaptive behavioral response mechanisms of animals to environmental changes. The diel activity rhythm of animals can reveal the spatiotemporal characteristics of energy metabolism, resource utilization, and survival strategies. Understanding the variation characteristics of animal activity rhythms is conducive to in-depth insights into their adaptive strategies to environmental changes, as well as analyzing the health status of individual animals and the availability of survival resources such as food and water in the environment [5]. Traditional studies on wildlife behavioral rhythms mostly rely on manual observation, which is limited by observation periods and sample sizes, leading to significant deviations in behavioral time calculation and restricting the accuracy of animal behavioral pattern analysis [6]. Infrared camera traps, characterized by small size, easy concealment, minimal interference with wildlife daily activities, and effective detection and recording of cryptic animal behaviors, enable all-weather monitoring and easy standardization. They have been widely used in wildlife surveys, providing an efficient and non-invasive solution for wildlife behavior research [7], particularly in studies on behavioral patterns and activity rhythms [8,9].

The daily activity patterns of wildlife are regulated by environmental factors [10]. Some wildlife species adjust their activity peaks to cope with predation, competition from sympatric species, and human activities. For example, coyotes (*Canis latrans*) and red foxes (*Vulpes vulpes*) modify their activity rhythms according to human activity levels in their habitats. However, such adjustments may have adverse effects, potentially reducing access to essential resources such as water and food, thereby altering relevant ecological processes [11,12,13]. Road traffic is one of the most prominent forms of human activity, and its impacts on wildlife and optimization strategies are important topics in conservation biology [14]. Road traffic can hinder wildlife movement routes, disrupt migration, induce avoidance behaviors, and even cause roadkill, threatening wildlife survival [15]. Nevertheless, wildlife can also adapt to human road traffic, utilizing roads to enhance movement convenience and foraging efficiency. For instance, leopard cats (*Prionailurus bengalensis*) prefer to be active along road edge habitats, as roads can improve movement connectivity and foraging efficiency to a certain extent [16]. Dickie and Serrouya (2018) demonstrated that road restrictions on caribou (*Rangifer tarandus caribou*) activity increased the foraging efficiency of sympatric predators such as wolves (*Canis lupus*) and American black bears (*Ursus americanus*) [17].

The Tibetan fox (*Vulpes ferrilata*) is an endemic species of the Qinghai–Tibet Plateau and a keystone species in the alpine ecosystem. Currently, due to the needs of human social development and construction, the habitats of Tibetan foxes are severely disturbed by human activities, resulting in habitat fragmentation and subsequent changes in their ecological behaviors [18,19]. In-depth understanding of their behavioral patterns and activity time allocation in response to human disturbance is a prerequisite for improving Tibetan fox conservation effectiveness. Previous studies have shown that juvenile Tibetan foxes exhibit a typical crepuscular activity pattern, resting mainly during the day, but their time allocation is also influenced by adult Tibetan foxes [20]. Tibetan foxes can achieve niche differentiation through behavioral adjustments with sympatric species such as Pallas’s cats (*Otocolobus manul*) and red foxes, enabling sympatric coexistence [21].

From the perspective of conservation veterinary medicine and animal welfare, wildlife behavioral patterns are not only ecological adaptation traits, but also intuitive external indicators of individual health status and population vitality. Changes in activity time allocation, anti-predation behavior and social communication behavior can reflect the level of physiological stress and energy load under environmental disturbance, which is of great value for early warning of population health risks and formulation of welfare-friendly conservation strategies. At present, the conservation veterinary implications of behavioral responses to human disturbance in plateau carnivores such as Tibetan foxes have not been fully discussed in related studies.

The Tangbei Area of Sanjiangyuan National Park is an important core distribution area for Tibetan foxes. However, to date, there is a lack of research on Tibetan foxes in this region, and the impacts of human activities such as road traffic on their behaviors remain unclear. Against this background, the present study aimed to answer two core scientific questions: (i) What are the behavioral structure and diel activity rhythm of Tibetan foxes in the Tangbei Area? (ii) How does road traffic disturbance affect their behavioral structure and activity allocation? Using infrared camera trapping, this study systematically quantified the behavioral structure, activity rhythm, and behavioral responses to road traffic of Tibetan foxes in the Tangbei Area, and integrated the perspectives of conservation veterinary medicine and animal welfare into the interpretation of these responses. In contrast to previous studies that mainly described general nocturnal activity and disturbance-avoidance patterns, this study further identified region-specific adaptive mechanisms, including the partial overlap between the activity peaks of Tibetan foxes and those of their main prey, the plateau pika, and the nocturnal activity strategy that helps to alleviate daytime traffic disturbance. The results provide important references for Tibetan fox conservation, human–wildlife conflict management, and ecological corridor optimization on the Qinghai–Tibet Plateau.

## 2. Materials and Methods

### 2.1. Study Area

The Tangbei Area of Sanjiangyuan National Park (hereinafter referred to as “Tangbei Area”), a core component of the Qinghai–Tibet Plateau ecological barrier, is a typical representative of global alpine ecosystems. It is the source of the Tuotuo River (the main headwater of the Yangtze River) and the Angqu River (a tributary of the Lancang River), known as the “life source of the Chinese Water Tower”. Located at the northern foot of the Tanggula Mountains at the junction of Qinghai and Tibet (32°43′~34°57′ N, 89°35′~95°20′ E), the Tangbei Area covers a total area of 48,700 km^2^, accounting for approximately 25.54% of the total area of Sanjiangyuan National Park. With an average elevation of 4600 m, the region features a typical plateau continental climate, with an annual average temperature of −4.2 °C and an average annual precipitation of 275.5 mm. Spanning Golmud City, Yushu Tibetan Autonomous Prefecture of Qinghai Province, and Anduo, Nierong, and Baqing Counties of Nagqu City, Tibet Autonomous Region, the area is under the actual management of the Tibet Autonomous Region.

### 2.2. Infrared Camera Monitoring

**Figure 1:**
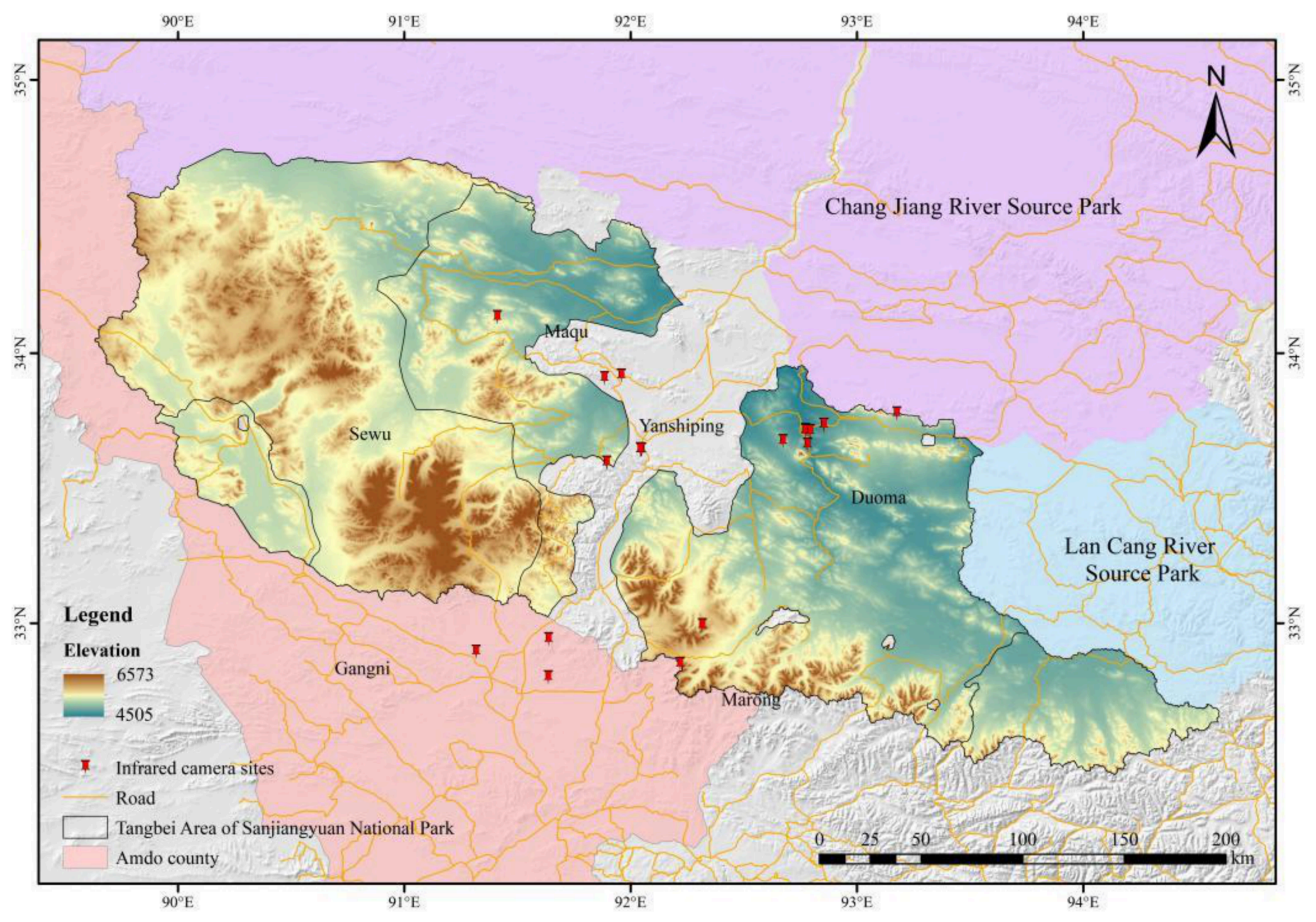
Locations of the Tangbei area of Sanjiangyuan National Park and infrared camera sites.

Considering factors such as vegetation integrity and representativeness, elevation, human activities, topography, and accessibility within the study area, and referring to the habitat use characteristics of Tibetan foxes [18,19], 401 infrared cameras were deployed from May to December 2024 in habitats preferred by Tibetan foxes, including alpine steppes, alpine meadows, small gully areas, mountain top bare rock areas, and flat grasslands adjacent to mountains (Figure 1). The distance between cameras was no less than 500 m. Cameras were fixed using stones and wooden stakes, and information such as camera ID, latitude, longitude, elevation, and habitat type of deployment sites was recorded. Camera sensitivity was set to “medium”, and the working mode was set to capture 3 photos and record a 20-s video upon triggering. Data storage cards and batteries were replaced every 3 months. Owing to the need to cover the habitats preferred by Tibetan foxes and to ensure the feasibility and accessibility of the field sites, the deployment followed a habitat-targeted rather than a strictly random sampling design.

### 2.3. Zoning of Road Traffic Disturbance Intensity

The straight-line distance between infrared camera deployment sites and major traffic roads (national highways and provincial highways) was used as an indicator to assess the intensity of road traffic disturbance. First, 1:1,000,000 vector data of major roads (including national and provincial highways) were downloaded from the National Geomatics Center of China (https://www.sgic.net.cn/portal/index.html#/Home accessed on 10 October 2025) and converted into raster surfaces, and the straight-line distance from each camera site to the nearest major road was calculated using the “Nearest Neighbor Analysis” tool based on the WGS84 latitude and longitude coordinates recorded during camera deployment. Analysis of the calculation results revealed that the number of infrared camera samples within 5 km of major roads was roughly equivalent to that beyond 5 km. Therefore, areas within 5 km of major roads were classified as high-disturbance zones, and areas beyond 5 km were classified as low-disturbance zones, completing the spatial zoning of disturbance intensity in the study area. All analyses were performed in ArcGIS 10.8. In this study, the straight-line distance to major roads was used as the sole quantitative indicator for defining the disturbance gradient; direct measurements of traffic flow, human activity frequency, and noise intensity were not included in the zoning.

### 2.4. Data Collation and Statistical Analysis

Camera memory cards were retrieved, and camera data were processed using Bio-Photo (V2.0) to batch extract information such as shooting date, time, and file type; match site IDs; and generate .csv format reports [7]. Memory cards capturing Tibetan fox images were screened, and the behaviors and durations of Tibetan foxes were sampled and recorded.

The ratio of the duration of a specific behavior of Tibetan foxes in each time period to the total sampling time was defined as the behavior occurrence ratio. The occurrence ratios of various behaviors of Tibetan foxes in different time periods were statistically analyzed. The normality of the behavioral data was first examined; the data for locomotor, vigilance, and scent-marking behavior showed a normal distribution (*p* > 0.05), whereas the data for other behaviors and the standardized data showed a non-normal distribution (*p* < 0.05). One-way ANOVA was used to compare the activity rates of Tibetan foxes among time periods, and independent-sample *t*-tests were used to compare the behavioral indicators (behavioral proportions and occurrence ratios) between the high- and low-disturbance zones, with the significance level set at *p* < 0.05.

## 3. Results

### 3.1. Behaviors and Ethogram of Tibetan Foxes

A total of 22 infrared camera sites capturing Tibetan fox images were selected for the study, accumulating 1166 effective camera trap days, 7260 photos (including 748 independent valid photos), 340 video clips, and 217 valid behavioral records of Tibetan foxes. Tibetan fox behaviors were classified into three basic behavioral categories (locomotor behavior, vigilance behavior, and scent-marking behavior) and other behaviors, with specific behaviors and their definitions as follows:

Locomotor Behavior: Including walking (slow alternating movement of limbs with stable steps, head usually horizontal or slightly lowered, typically for daily movement and territory patrol), trotting (brisk steps with diagonal movement of forelimbs and hindlimbs, moderate speed, typically for long-distance movement and environmental exploration), running (rapid sprinting with quick alternating limb contact with the ground, obvious off-ground phase, tail extended horizontally or slightly raised, typically for escaping natural enemies such as wolves, snow leopards, and birds of prey, and chasing prey), and jumping (leaping into the air with hindlimb force, common for crossing obstacles or capturing prey such as pikas, typically for foraging or terrain adaptation).

Vigilance Behavior: Including freezing (sudden stillness during running or walking, rigid body with only slight eye and ear movements to detect environmental information), alert stand (erect limbs, tense body, tail hanging down or slightly raised, as a defensive preparation in high-risk environments), and scanning (standing or squatting and staring, head raised, ears rotating, gaze fixed on a specific direction or scanning back and forth, typically for monitoring threats from natural enemies or conspecifics).

Scent-Marking Behavior: Mainly scent marking (sniffing the ground, urinating, and rubbing the body surface or tail (e.g., anal glands) against rocks, vegetation, or other objects, used for territory declaration, information recognition, or information transmission).

Other Behaviors: Due to the extremely low frequency of behaviors such as excretion and social interactions recorded by infrared cameras in this study, these behaviors were collectively classified as other behaviors.

### 3.2. Behavioral Composition and Temporal Allocation Pattern of Tibetan Foxes

**Figure 2:**
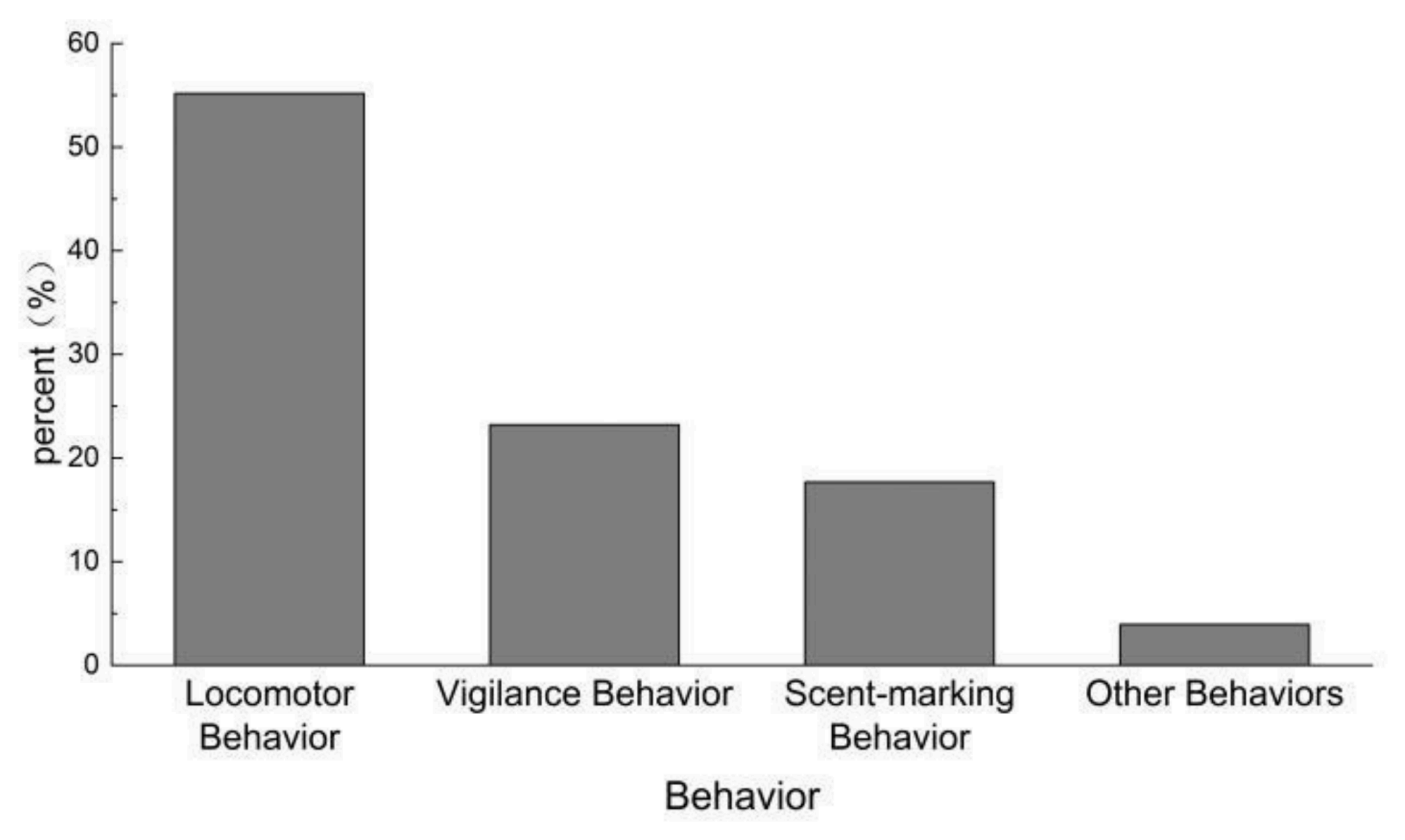
Behavioral duration of wild Tibetan foxes.

After collating and summing up Tibetan fox behavioral data, the proportion of total duration of each behavioral category was determined, as shown in Figure 2. Locomotor behavior accounted for the highest proportion (55.18%), followed by vigilance behavior (23.20%), scent-marking behavior (17.68%), and other behaviors (3.94%).

The data of locomotor, vigilance, and scent-marking behaviors of Tibetan foxes showed a normal distribution (*p* > 0.05), while the data of other behaviors and standardized data showed a non-normal distribution (*p* < 0.05).

**Figure 3:**
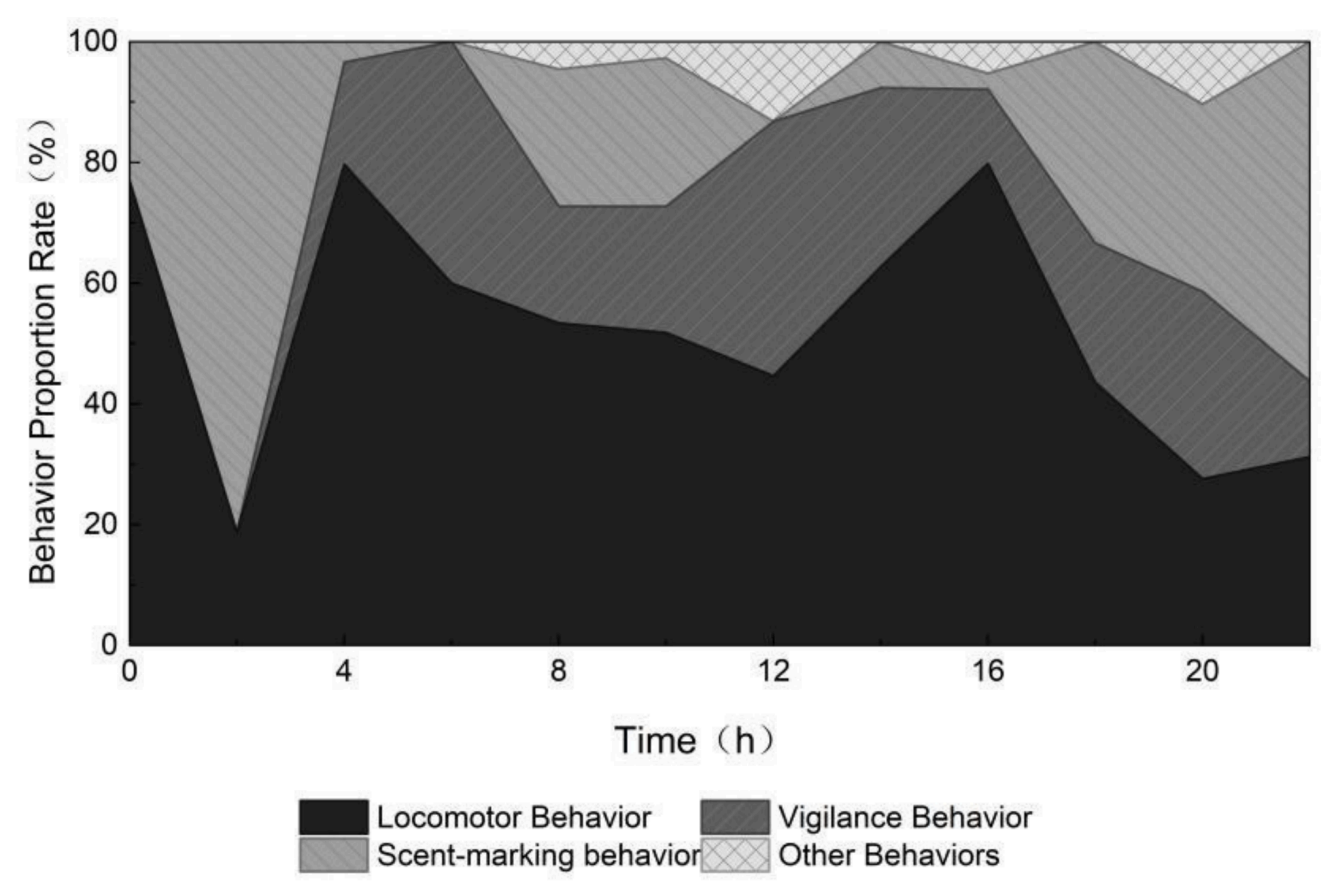
Distribution of behavior duration of Tibetan fox.

The temporal distribution of the occurrence ratio of each behavior of Tibetan foxes is shown in Figure 3. The occurrence ratio of locomotor behavior was the highest (52.4% ± 5.88%; n = 132; *p* = 0.783 > 0.05), followed by vigilance behavior (20.6% ± 3.92%; n = 54; *p* = 0.977 > 0.05) and scent-marking behavior (23.8% ± 7.16%; n = 26; *p* = 0.318 > 0.05), while other behaviors had the lowest occurrence ratio (3% ± 1.32%; n = 6; *p* = 0.001 < 0.05), where the *p* values denote the results of normality tests.

### 3.3. Activity Peaks of Tibetan Foxes

**Figure 4:**
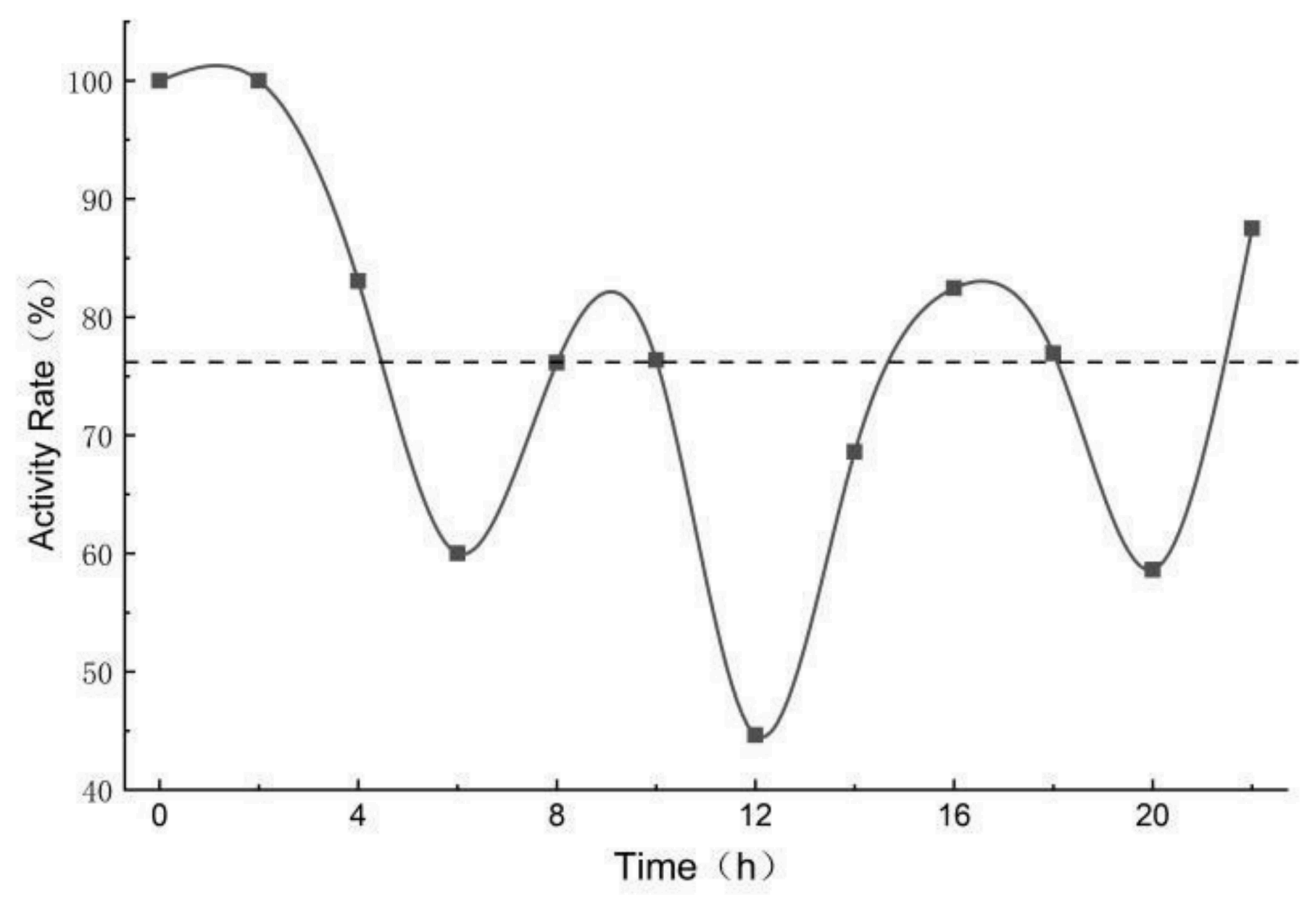
Distribution of activity rate and activity peak of wild Tibetan fox.

The locomotor and scent-marking behaviors of Tibetan foxes were summed to characterize their active state. As shown in Figure 4, the average daily activity rate of Tibetan foxes was 76.20% (±4.75%).

Using the mean activity rate as the baseline for dividing activity peaks, Tibetan foxes exhibited an obvious long-duration nighttime activity peak (22:00–04:00) with an activity rate of 92.63%, a small afternoon activity peak (16:00) with an activity rate of 82.45%, and low activity rates in the morning (6:00), noon (12:00), and evening (20:00). Among these, the nighttime activity peak (22:00–04:00) lasted for a relatively long time (6 h) with an activity rate of 92.63%, while the activity rate at noon (12:00) reached the lowest value of the day (44.63%).

### 3.4. Impacts of Road Traffic on the Behavior of Tibetan Foxes

**Figure 5:**
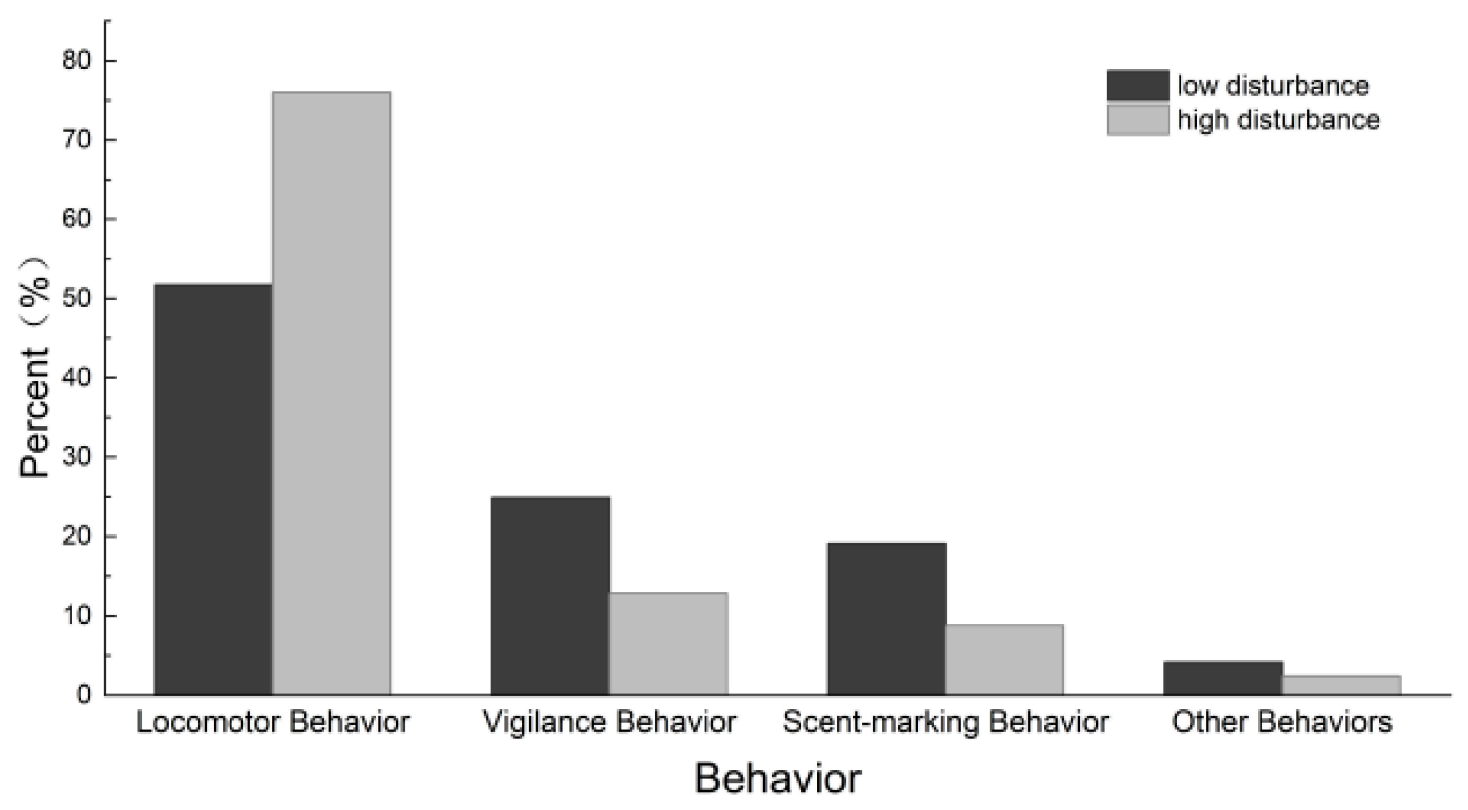
Behavioral composition of Tibetan fox in areas with high and low disturbance.

As shown in Figure 5, in areas with high road traffic disturbance, Tibetan foxes exhibited more locomotor behavior (76.00%) with a mean occurrence rate of 61.93% (±12.18%); vigilance behavior accounted for 12.80% with a mean occurrence rate of 17.16% (±9.30%); and scent-marking behavior accounted for 8.80% with a mean occurrence rate of 10.91% (±7.36%). The average daily activity rate of Tibetan foxes in high-disturbance areas was 72.84% (±11.88%).

In areas with low road traffic disturbance, locomotor behavior also accounted for the highest proportion (51.76%) with a mean occurrence rate of 51.57% (±6.22%); vigilance behavior accounted for 24.9% with a mean occurrence rate of 21.87% (±3.83%); and scent-marking behavior accounted for 19.13% with a mean occurrence rate of 23.48% (±7.21%). The average daily activity rate of Tibetan foxes in low-disturbance areas was 75.04% (±4.48%).

A total of 23 behavioral records of wild Tibetan foxes were obtained in high-disturbance areas, which was less than the 194 records in low-disturbance areas, indicating that Tibetan foxes prefer to be active in areas with low road traffic disturbance. Independent-sample *t*-tests showed that the proportion of locomotor behavior of Tibetan foxes in high-disturbance areas (76.00%) was significantly higher than that in low-disturbance areas (51.76%) (*p* < 0.05), while the proportion of scent-marking behavior in low-disturbance areas (19.13%) was significantly higher than that in high-disturbance areas (8.80%) (*p* < 0.05).

## 4. Discussion

### 4.1. Behavioral Pattern and Ecological Adaptation of Tibetan Foxes in the Tangbei Area of Sanjiangyuan National Park

This study found that the behaviors of Tibetan foxes in the Tangbei Area of Sanjiangyuan National Park are mainly composed of locomotor behavior (55.18%), vigilance behavior (23.20%), and scent-marking behavior (17.68%). This is highly consistent with its trophic niche characteristics as a predator. As a predatory species, the foraging, territory patrolling, and environmental exploration of Tibetan foxes are all accompanied by locomotor behavior. The high occurrence rate of vigilance behavior (23.20%) and frequent display of behaviors such as freezing (natural stillness), standing and staring, and environmental exploration reflect the high anti-predation and prey-searching needs of Tibetan foxes, which is consistent with the common vigilance and defense strategies of small carnivores [22]. The stable proportion of scent-marking behavior indicates that Tibetan foxes in the Tangbei Area of Sanjiangyuan National Park rely heavily on chemical communication to maintain territory boundaries and transmit intraspecific information. This behavioral pattern is a typical social characteristic of Canidae. For example, North American red foxes use chemical communication methods such as urine marking for territory declaration [23], whose ecological function is similar to the scent-marking behavior of Tibetan foxes in this study.

From the view of conservation veterinary medicine, the ethogram and time budget established in this study provide a baseline behavioral reference for wild Tibetan foxes in the Tangbei Area. This baseline can be used as a comparative standard for future population health monitoring, and also provides a quantitative basis for welfare assessment of captive Tibetan fox populations, helping to identify abnormal behaviors caused by stress or health problems.

It is worth noting that no foraging behavior of Tibetan foxes was captured in this study. This absence is probably attributable to a combination of sampling characteristics and ecological factors. Regarding sampling characteristics, the camera sites were mainly placed in habitats preferred by Tibetan foxes, such as alpine steppes and meadows, where passing, patrolling, and vigilance behaviors are more likely to be recorded, whereas foraging may occur at specific foraging patches that were not effectively covered by the cameras; moreover, the working mode of the cameras (three photos and a 20-s video per trigger) may not capture the relatively stationary and prolonged foraging process. Regarding ecological factors, the concealment of Tibetan fox foraging behavior itself, as well as possible regional food scarcity and an extremely low foraging success rate, may further reduce the probability of foraging being recorded; if food resources are indeed limited, this may restrict the survival of Tibetan foxes. In future studies on Tibetan fox behavior, the habitat coverage of camera deployment should be optimized to include potential foraging patches and combined with dietary research techniques such as fecal analysis, and comprehensive studies on the foraging behavior of Tibetan foxes should be conducted to evaluate the nutritional stress level of Tibetan foxes in the region.

### 4.2. Activity Allocation and Behavioral Rhythm of Tibetan Foxes in the Tangbei Area of Sanjiangyuan National Park

This study showed that the diel activity rhythm of Tibetan foxes in the Tangbei Area of Sanjiangyuan National Park exhibits significant temporal differentiation, with an obvious nighttime activity peak (22:00–04:00, activity rate 92.63%), a small afternoon peak (16:00, activity rate 82.45%), and a noon trough (12:00, activity rate 44.63%). This indicates that the activity pattern of Tibetan foxes is dominated by nocturnality, accompanied by crepuscular activity peaks. This behavioral rhythm pattern is an adaptive manifestation driven by multiple factors, reflecting the adaptive strategy of Tibetan foxes to the alpine environment.

As one of the flagship species in the typical alpine ecosystem of the Qinghai–Tibet Plateau, low temperature is the most severe environmental stress factor for Tibetan foxes. Their thick fur provides excellent cold resistance, laying a physiological foundation for their high activity at night. From the perspective of food resource utilization, the main prey species of Tibetan foxes, the plateau pika (Ochotona curzoniae), exhibits high crepuscular activity [24] and has a daily activity peak around 16:00 [25]. The results of this study show that the activity pattern of Tibetan foxes in the Tangbei Area of Sanjiangyuan National Park—dominated by nighttime activity and accompanied by crepuscular activity peaks—enables partial overlap between the activity peak of Tibetan foxes and that of pikas in the region. The partial overlap between the activity peaks of Tibetan foxes and pikas in this study is a unique ecological process formed by the long-term coevolution of the “predator–prey system” in the Qinghai–Tibet Plateau ecosystem, which not only ensures the food resources of Tibetan foxes but also prevents the population collapse of pikas due to predation by Tibetan foxes.

Regarding the discrepancy between the crepuscular activity pattern reported for juvenile Tibetan foxes in a previous study [20] and the predominantly nocturnal activity of adults observed in this study, several explanations can be proposed. First, age-related differences in predation risk and thermoregulatory requirements may play a role: juveniles resting at dens are relatively sheltered and less exposed to daytime predation risk and heat stress, whereas free-ranging adults, which need to move over long distances and patrol territories, may be more strongly constrained by daytime human activities and traffic disturbance, which favors nocturnal activity. Second, adults may trade off their activity timing with the activity rhythms of their prey, because the crepuscular components of the adult activity pattern partially overlap with the activity peaks of the plateau pika. Third, methodological differences between studies, namely direct observation of pups at den sites versus infrared camera trapping of adults, may also contribute to the observed discrepancy. These hypotheses require further testing with individual-based behavioral data.

In addition, the behavioral rhythm characteristics of Tibetan foxes are also an important manifestation of interspecific niche differentiation in the alpine ecosystem. The long-duration nighttime activity peak of Tibetan foxes shows significant temporal mismatch with sympatric closely related species such as red foxes, with extremely low overlap of their daily activity peaks [26,27]. This temporal segregation is consistent with a previous study in the source region of the Yangtze River, which documented spatiotemporal overlap among sympatric Pallas’s cats (*Otocolobus manul*), Tibetan foxes, and red foxes and indicated that these species reduce interspecific competition through temporal and spatial niche partitioning [21]. This indicates that species in the same resource guild reduce competition for food resources through temporal niche partitioning. A similar strategy is observed in medium-sized carnivores in Wolong Nature Reserve, which achieve sympatric coexistence through dual differentiation of habitat use and activity periods [28,29].

From the perspective of animal welfare, this nocturnal-dominant activity pattern with crepuscular peaks is the result of the adaptive trade-off between foraging demand, predation risk and environmental stress. The maintenance of natural activity rhythm is an important manifestation of good welfare status for wild populations, and the destruction of this rhythm by human disturbance will trigger a series of health risks such as endocrine disorder and reduced immunity.

### 4.3. Impacts of Road Traffic Disturbance on the Behavioral Pattern of Tibetan Foxes

With the advancement of ecological civilization construction on the Qinghai–Tibet Plateau, the state has increased investment in infrastructure for ecological management in the Tangbei Area of Sanjiangyuan National Park, optimizing regional road traffic. In addition, the region has a relatively large number of towns and herder settlements, and the “village-to-village connection” project has ensured transportation and living convenience between herder settlements. On the other hand, road traffic in the Tangbei Area of Sanjiangyuan National Park may have impacts on wildlife in the region.

This study showed that the number of behavioral records of Tibetan foxes in low-road-traffic-disturbance areas (194 times) is much higher than that in high-disturbance areas (23 times), directly reflecting the avoidance tendency of Tibetan foxes towards human activity disturbance, which is consistent with the behavioral response strategies of other wildlife [30]. The Tibetan foxes in the region exhibit an obvious nighttime activity peak accompanied by crepuscular activity peaks. In addition to adapting to food acquisition, this is also an avoidance response of Tibetan foxes to the high daytime traffic volume. The avoidance tendency of Tibetan foxes towards road-dense areas complements their nighttime activity peak, as they avoid periods of high human activity through activity peak separation, reducing stress from human disturbances such as road traffic.

This study confirmed that road traffic in the Tangbei Area of Sanjiangyuan National Park has a significant impact on the behavioral allocation pattern of Tibetan foxes. The proportion of locomotor behavior of Tibetan foxes in areas with high road traffic intensity (61.93%) is significantly higher than that in low-disturbance areas (51.76%), while the occurrence rate of scent-marking behavior is significantly lower (8.80%). This indicates that under the influence of road traffic, Tibetan foxes increase the time allocated to locomotor behavior for foraging, patrolling, and movement while reducing intraspecific social interactions such as chemical marking.

Firstly, noise pollution from roads and accompanying high-intensity human activities (e.g., direct entry into the habitat of Tibetan foxes) can directly trigger acute stress responses in Tibetan fox [31], leading to frequent adjustments of their movement paths to avoid potential threats. Such behavioral adjustments disrupt the normal foraging and resting cycles of Tibetan foxes, increase individual energy metabolism and consumption, indirectly reduce their energy reserves and investment in cold resistance, and ultimately affect their fitness.

From the perspective of conservation veterinary medicine, this increased energy expenditure and disrupted behavioral rhythm represent a potential chronic stress state. Long-term exposure to such disturbance may lead to sustained elevation of glucocorticoid levels, suppressed immune function, and reduced reproductive success rate, which are typical health risks of wildlife under anthropogenic pressure. The significant reduction in scent-marking behavior not only affects social structure, but also means that the natural expression of species-specific social behavior is restricted, which is an important indicator of reduced animal welfare. The impacts of road traffic on wildlife have been verified in many studies on wildlife species [10].

Secondly, road traffic in wildlife habitats may cause habitat fragmentation through barrier effects, thereby affecting the survival of wildlife [32]. The results of this study show that Tibetan foxes in the Tangbei Area of Sanjiangyuan National Park exhibit more locomotor behavior and less scent-marking behavior in areas with high traffic intensity, indicating that changes in their behavioral pattern are related to the impacts of road traffic. Road traffic weakens the continuity and connectivity of Tibetan fox habitats, leading to increased locomotor duration of Tibetan foxes in this study to search for passages, cross barriers, and move to edge habitats. This adjustment of behavioral strategies increases the movement energy consumption of Tibetan foxes, reduces their foraging time and energy reserves, and disturbs the habitat and territory stability of Tibetan fox populations in the region. The population regulation of Canidae highly depends on chemical communication between individuals. Scent marking by individuals can transmit information about reproductive status, territory boundaries, and individual fighting ability. The weakening of marking behavior will reduce the efficiency of information transmission, leading to reproductive failure and increased intraspecific conflicts [33]. In this study, the reduction or absence of scent-marking behavior in Tibetan fox individuals may affect their reproduction and social structure, ultimately influencing their individual fitness and population growth. Collectively, these disturbance-induced behavioral changes may affect population diffusion by altering movement patterns, disrupt social communication by reducing scent-marking, lower reproductive success through weakened information transfer and increased intraspecific conflicts, and raise energy expenditure at the expense of the individual energy budget, ultimately influencing population dynamics and long-term viability.

These behavioral response characteristics can be used as non-invasive monitoring indicators for the conservation veterinary assessment of Tibetan fox populations. Compared with invasive physiological sampling, behavioral monitoring based on infrared cameras is more suitable for long-term health assessment of rare plateau carnivores, and can realize early warning of population health risks before obvious physiological damage occurs. Furthermore, through the trophic cascade effect in the food chain, it may affect the survival of other species such as plateau pikas, thereby reducing the biodiversity level in the sampled area.

Taken together, the findings of this study can be systematically integrated into a conservation veterinary medicine and animal welfare framework. First, the quantified behavioral baseline (behavioral composition, activity rhythm, and disturbance-induced behavioral shifts) provides reference indicators for monitoring the health status of wild Tibetan fox populations and for welfare assessment of captive populations. Second, behavioral responses such as increased locomotion, reduced scent-marking, and disrupted activity rhythm can serve as early, non-invasive warning signals of chronic stress and reduced welfare before obvious physiological damage occurs. Third, integrating behavioral monitoring with veterinary health assessments and physiological stress biomarkers would improve the early warning and adaptive management of health risks in plateau carnivores under increasing anthropogenic pressure.

### 4.4. Suggestions for Ecological Resource Conservation and Management

To strengthen the conservation of Tibetan foxes in the Tangbei Area of Sanjiangyuan National Park from the perspectives of conservation veterinary medicine and animal welfare, the following suggestions are proposed:

(1) Implement time-sharing traffic control and construct buffer zones to reduce disturbance-induced welfare damage. Given that Tibetan foxes in the Tangbei Area exhibit a pronounced nocturnal activity peak (22:00–04:00), a secondary afternoon activity peak (around 16:00), and a clear tendency to avoid high-disturbance areas, we recommend (i) implementing traffic restriction or speed-limit measures during the afternoon activity peak (15:30–16:30) and, where feasible, reducing traffic intensity and noise-generating activities (e.g., road maintenance and lighting) during the nocturnal activity peak, and (ii) constructing “shrub + herb” buffer zones in road sections with high Tibetan fox crossing frequency to buffer noise and visual disturbance. These measures can reduce the stress response and additional energy consumption of Tibetan foxes caused by traffic flow.

(2) Construct wildlife passages and ecological corridors to ensure habitat connectivity. The design of corridors should fully consider the nocturnal movement and territory-maintenance behavior of Tibetan foxes, minimize the barrier effect of roads on movement and territory maintenance, and, given that scent-marking, a key channel of social communication, was markedly reduced in high-disturbance areas, ensure that corridors and their adjacent habitats maintain sufficient connectivity for the exchange of scent-marking information among individuals. These measures will help avoid the decline of population health caused by long-term habitat fragmentation and obstructed social communication.

(3) Establish a long-term monitoring system integrating behavior and conservation veterinary medicine. Take the behavioral indicators identified in this study (locomotion ratio, scent-marking frequency, activity rhythm deviation) as routine monitoring indicators, and regularly evaluate the welfare status and health risk of Tibetan fox populations under the background of increasing human activities so as to realize adaptive management of protected areas.

## Data Availability

The raw data supporting the conclusions of this article will be made available by the authors on request.
